# Double bare metal stent deployment combined with intraductal radiofrequency ablation for malignant distal biliary obstruction: a prospective pilot study

**DOI:** 10.1038/s41598-023-29955-5

**Published:** 2023-02-14

**Authors:** Tadahisa Inoue, Mayu Ibusuki, Rena Kitano, Kazumasa Sakamoto, Satoshi Kimoto, Yuji Kobayashi, Yoshio Sumida, Yukiomi Nakade, Kiyoaki Ito, Masashi Yoneda

**Affiliations:** Department of Gastroenterology, Aichi Medical University, 1-1 Yazakokarimata, Nagakute, Aichi 480-1195 Japan

**Keywords:** Gastroenterology, Therapeutics

## Abstract

Although uncovered self-expandable metal stents (SEMSs) possess certain advantages such as averting cystic duct obstruction and stent migration, they are susceptible to ingrowth occlusion. The combination of the double bare stent (DBS) and endobiliary radiofrequency ablation (RFA) may reduce ingrowth. Hence, this study aimed to examine the utility of this method for the treatment of unresectable malignant distal biliary obstruction (MDBO). This prospective, single-center, pilot study enrolled 51 patients who met the eligibility criteria between February 2020 and January 2022. The study outcomes included technical success, clinical success, recurrent biliary obstruction (RBO), and other adverse events (AE) besides RBO associated with DBS placement with RFA for MDBO. The technical success rate was 98.0% (50/51). Clinical success was achieved in all patients in whom technical success was achieved. The rates of early and late AEs were 5.9% (3/51) and 8.0% (4/50), respectively. The incidence rate of RBO was 38.0% (19/50). Sludge occlusion, ingrowth occlusion, and overgrowth occlusion occurred in 26.0% (13/50), 8.0% (4/50), and 2.0% (1/50) of patients, respectively (the main cause of RBO was undeterminable in 1 patient). The median time to RBO was 241 days. DBS with RFA showed good technical feasibility, good long-term outcomes, acceptable AE rates, and most importantly, a low ingrowth occlusion rate when employed for the treatment of MDBO.

## Introduction

Endoscopic self-expandable metal stent (SEMS) deployment is recommended for unresectable malignant distal biliary obstruction (MDBO) because it offers a longer patency period compared to plastic stent placement^1^. The SEMS can be categorized into the uncovered SEMS and covered SEMS^2^, each of which has advantages and disadvantages; however, the superiority of one system over the other remains inconclusive^1,3,4^. In the current scenario, the prevalence of recurrent biliary obstruction (RBO) is relatively high, irrespective of the type of stent used, owing to the advancement in antitumor therapy. Recently, a uniquely structured SEMS, which is constructed by the cross-overlapping of two uncovered SEMS called the “double bare stent (DBS),” has been developed with the aim to extend the patency period even further^5^. This unique SEMS is expected to reduce ingrowth, which is a major cause of uncovered SEMS occlusion, by reducing the size of the stent cells.

On the other hand, endobiliary radiofrequency ablation (RFA), which has been reported to prolong the patency period, has recently emerged as an adjuvant procedure during biliary stent placement^6^. It is assumed that RFA can reduce ingrowth by inducing coagulative necrosis of the tumor tissue in the strictures. Therefore, the combination of DBS and RFA may further prevent ingrowth, which prolongs patency, while retaining the advantages of the uncovered SEMS. This study aimed to examine the feasibility and utility of this combination treatment with DBS and RFA in patients with MDBO.

## Methods

### Study design and population

This prospective, single-center, pilot study included consecutive patients aged 20 years or older who developed obstructive jaundice and/or cholangitis due to unresectable MDBO between February 2020 and January 2022. The exclusion criteria were as follows: difficulty in reaching the duodenal papilla, surgically altered gastrointestinal anatomy, short stricture length unsuitable for the RFA catheter used in the study^7^, Eastern Cooperative Oncology Group performance status 4, severe dysfunction in any other organ, and refusal to participate in the study. Temporary predrainage was implemented with a plastic stent or nasobiliary drainage until the presence of a malignancy and unresectability, such as metastasis, locally advanced, and/or poor general condition were confirmed.

The institutional review board of Aichi Medical University Hospital approved this study, which was conducted in accordance with the principles of the Declaration of Helsinki (approval number: 2019-169). All patients provided written informed consent before the procedure and study participation. The study protocol was registered with the University Hospital Medical Information Network Clinical Trial Registry database (Identifier: UMIN000039563).

### Devices

**Figure 1:**
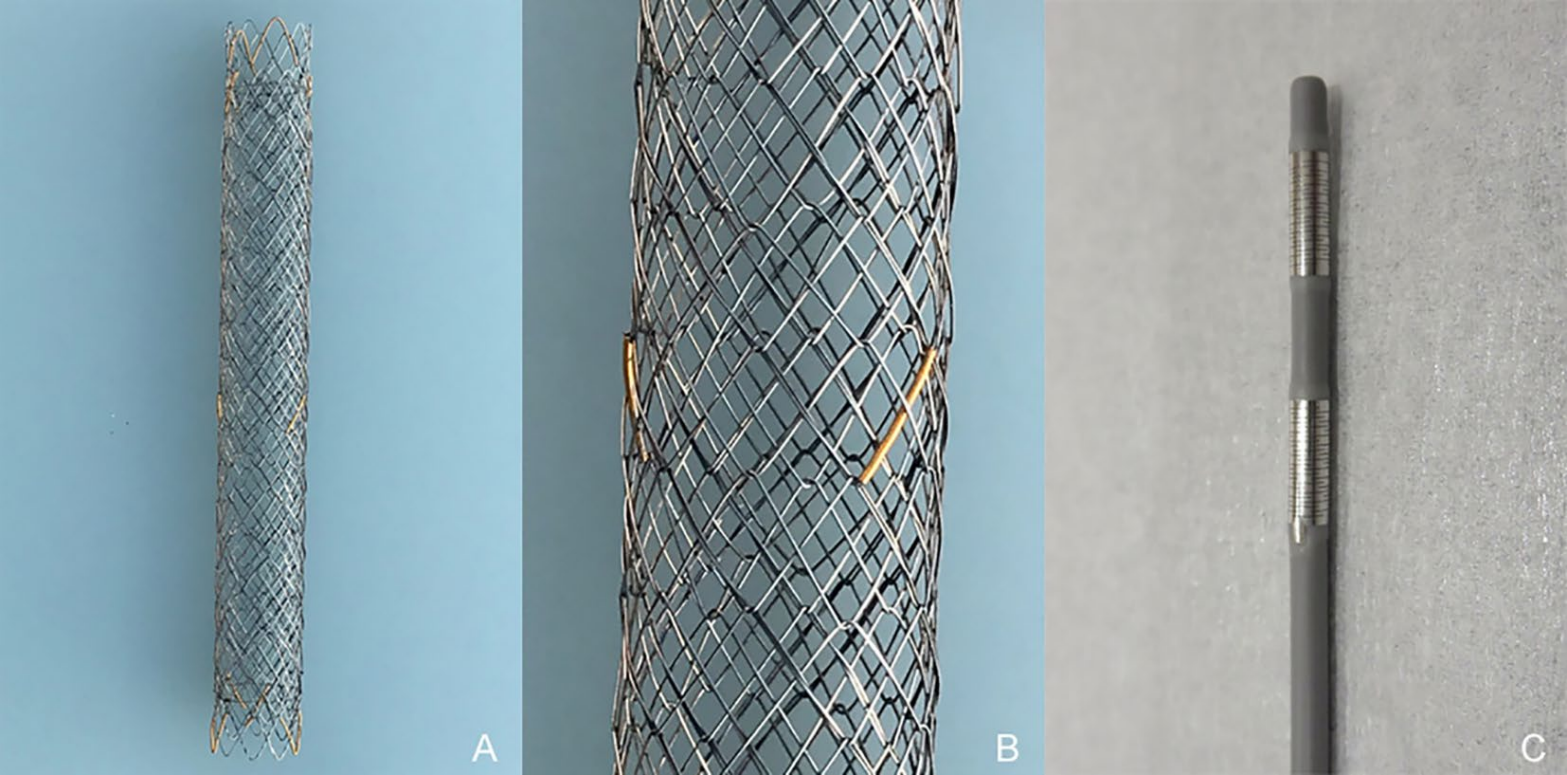
The structure of the EGIS biliary double bare stent (S&G Biotech, Seoul, Korea) is composed of two cross-overlapping standard uncovered stents, which can reduce the cell size and ensure sufficient radial force (**A,B**). The Habib EndoHPB catheter (Boston Scientific, Marlborough, MA, USA) is a bipolar catheter specializing in endobiliary radiofrequency ablation with two 8-mm ring electrodes placed 8-mm apart at the catheter tip (**C**).

The EGIS biliary DBS (S&G Biotech, Seoul, Korea) and the Habib EndoHPB catheter (Boston Scientific, Marlborough, MA, USA) were used for the biliary drainage procedure in this study (Fig. 1). The DBS is composed of two cross-overlapping standard uncovered stents. The overlap creates smaller stent cells and generates sufficient radial force. A 10-mm diameter stent was used for all cases, and the stent length was chosen according to the stricture length. The Habib bipolar catheter is composed of two 8-mm ring electrodes placed 8-mm apart at the catheter tip. The tissues surrounding the catheter are ablated when the electrodes come into proper contact with the stricture.

### Procedure

**Figure 2:**
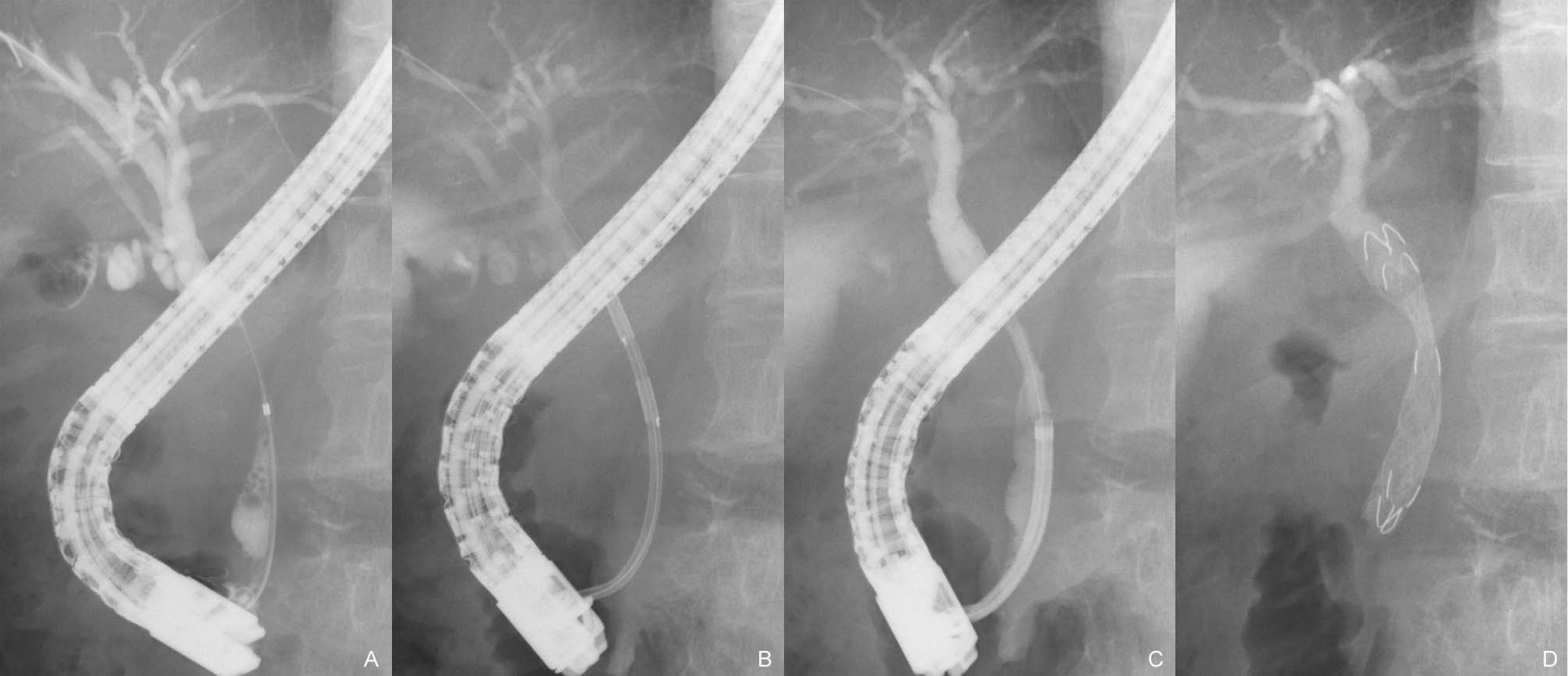
Severe stricture of the distal bile duct (**A**). Ablation of the stricture was performed (**B**), which improved the stricture diameter (**C**). A double bare stent is subsequently placed to cover the stricture (**D**).

The procedure was performed in the prone position under conscious sedation using midazolam with pethidine. A standard side-viewing duodenoscope (Olympus Medical Systems, Tokyo, Japan) was inserted into the duodenum. Biliary cannulation and cholangiography were performed, followed by insertion of the Habib catheter over the guidewire, which was positioned with the electrodes in contact with the stricture site. Subsequently, the stricture was ablated at a power of 7–10 W for 90 s using a VIO300D generator (ERBE Elektromedizin GmbH, Tübingen, Germany). RFA application was repeated until the entire stricture was ablated, if the length of the stricture was greater than the scope of ablation with a single application. After ablation, the bile duct was cleaned sufficiently with a retrieval balloon and/or basket catheter. Finally, the DBS was inserted and deployed to cover the stricture (Fig. 2 and Video 1). Endoscopic sphincterotomy was performed in all cases.

In the event of RBO, transpapillary reintervention was attempted initially. First, the inner aspects of the DBS and bile duct were swept sufficiently with a retrieval balloon catheter. If recanalization was good, the primary reason for RBO was judged to be sludge formation, and the procedure was completed using balloon cleaning alone. If good recanalization could not be obtained after balloon cleaning, the primary reason for RBO was judged to be ingrowth with/without overgrowth (persistent stricture located within the stent was ingrowth, and persistent stricture located below or above the stent was overgrowth), and additional stent placement was performed with/without RFA.

An endoscopist who was experienced in biliary stenting and endobiliary RFA procedures conducted or directly supervised all procedures, including initial stenting and reintervention.

### Follow-up

All patients were followed-up after the procedure using clinical examinations and laboratory tests at least once every 4 weeks until death or transfer to another institution. Imaging studies, including computed tomography, were performed when adverse events (AE), including RBO, were suspected. Computed tomography was conducted every 2–3 months to evaluate disease progression in patients who underwent chemotherapy, even in those without suspected AEs.

### Outcomes and definitions

The study outcomes included technical success, clinical success, RBO, and other AEs besides RBO that were associated with DBS placement with RFA for MDBO.

Technical success was defined as the successful completion of biliary cannulation, RFA catheter insertion, stricture ablation, and DBS placement. Clinical success was defined as the decrease in bilirubin and liver enzyme levels to normal or < 50% of preoperative levels within 14 days. RBO was defined as the recurrence of jaundice and/or cholangitis accompanied by biliary dilation on imaging studies^8^. The time to RBO was defined as the period from the date of DBS placement with RFA to the date of occurrence of RBO. AEs (except RBO) were defined and graded according to the criteria provided by the American Society for Gastrointestinal Endoscopy^9^.

### Statistical analysis

Categorical variables were expressed as numbers and percentages, and continuous variables were expressed as means and standard deviation (SD). The Kaplan–Meier method was used to estimate the time to RBO. The data of patients who did not develop RBO until the time of death, transfer to another institution, or the end of the study period were censored. All statistical analyses were conducted using EZR version 1.54 (Saitama Medical Center, Jichi Medical University, Saitama, Japan)^10^. P-values < 0.05 were considered statistically significant.

## Results

### Patient characteristics

Fifty-one patients who met the eligibility criteria were enrolled in this study. Table 1 presents the patients’ characteristics, including sex, age, etiology, bilirubin level, presence of cholangitis, stricture status, presence of concomitant duodenal stricture, and chemotherapy status. The median follow-up period was 172 (IQR, 80–280) days.

**Table 1:** Baseline characteristics of the participants.

Number of patients, N	51
Sex, (male/female), n	26/25
Mean age (SD), years	77.0 (10.8)
Etiology, n (%)	
Pancreatic cancer	40 (78.4)
Bile duct cancer	4 (7.8)
Gallbladder cancer	2 (3.9)
Others	5 (9.8)
Mean bilirubin level (SD), mg/dL	6.0 (5.8)
Presence of cholangitis, n (%)	18 (35.3)
Mean length of the stricture (SD), mm	21.2 (6.2)
Concomitant duodenal stricture, n (%)	15 (29.4)
Received chemotherapy	29 (56.9)
Mean follow-up period (SD), days	203.3 (169.2)

*SD* standard deviation.

### Outcomes

Table 2 presents the outcomes of DBS placement combined with RFA. The technical success rate was 98.0% (50/51), and biliary cannulation failed in 1 patient. The median operative time was 18 (IQR, 15–22) min. Clinical success was achieved in all patients in whom technical success was achieved.

**Table 2:** Outcomes of double bare metal stent deployment combined with radiofrequency ablation for malignant distal biliary obstruction.

Technical success, n (%)	50/51 (98.0)
Length of stent, n	
50 mm	6
60 mm	13
70 mm	23
80 mm	8
Deployment method, n	
Across the papilla	44
Above the papilla	6
Mean operative time (SD), min	20.3 (8.9)
Clinical success, n (%)	50/50 (100)
Early adverse events besides RBO (≤ 30 days), n (%)	3/51 (5.9)
Pancreatitis	1 (2.0)
Cholecystitis	1 (2.0)
Cholangitis	1 (2.0)
Late adverse events besides RBO (≥ 31 days), n (%)	4/50 (8.0)
Hemobilia	2 (4.0)
Liver abscess	1 (2.0)
Non-occlusion cholangitis	1 (2.0)
Incidence of RBO, n (%)	19/50 (38.0)
Primary causes of RBO, n (%)	
Sludge formation	13 (26.0)
Ingrowth	4 (8.0)
Overgrowth	1 (2.0)
Unknown/undeterminable	1 (2.0)
Successful endoscopic transpapillary reintervention for RBO, n (%)	18/19 (94.7)
Method of transpapillary reintervention, n	
Only balloon cleaning	13
Additional stent placement	5
Median time to RBO (95% CI), days	241 (134-NA)
90-day non-RBO rate (95% CI), %	79.9 (63.7–89.5)
180-day non-RBO rate (95% CI), %	60.5 (42.1–74.7)
Median overall survival (95% CI), days	175 (115–281)
90-day survival rate (95% CI), %	75.8 (61.3–85.4)
180-day survival rate (95% CI), %	48.6 (34.0–61.8)

*SD* standard deviation, *RBO* recurrent biliary obstruction, *CI* confidence interval, *NA* not applicable.

The rate of early AEs was 5.9% (3/51), which included mild pancreatitis (n = 1), mild cholangitis (n = 1), and moderate cholecystitis (n = 1), necessitating endoscopic ultrasound-guided gallbladder drainage. The rate of late AEs was 8.0% (4/50). Non-occlusion cholangitis, which occurred in 1 patient, improved with conservative management. One patient developed a liver abscess, which required percutaneous drainage. Hemobilia occurred in 2 patients, one of whom was treated with conservative management and the other required interventional radiology.

**Figure 3:**
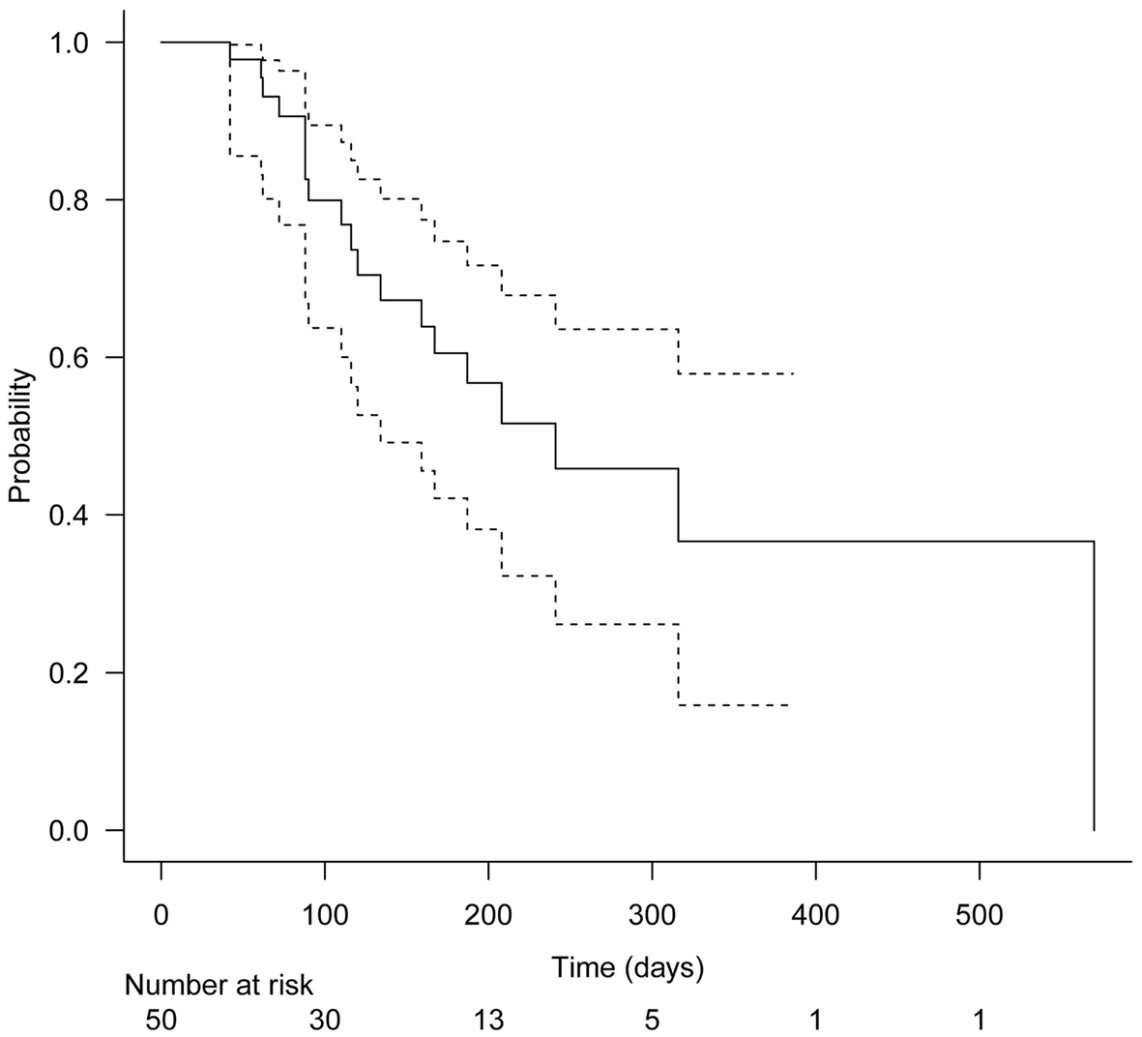
Kaplan–Meier analysis of the time to recurrent biliary obstruction. The median time to recurrent biliary obstruction was 241 days (95% confidence interval, 134-not applicable) days.

**Figure 4:**
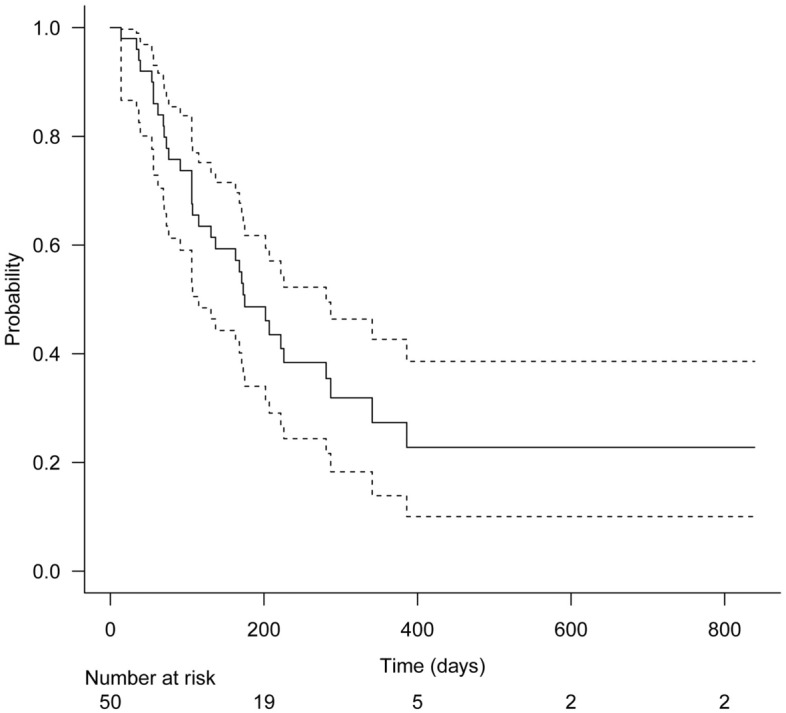
Kaplan–Meier analysis of overall survival. The median overall survival was 175 (95% confidence interval, 115–281) days.

The incidence rate of RBO was 38.0% (19/50). The primary causes of RBO were sludge formation in 13 patients, ingrowth in 4 patients, overgrowth in 1 patient, and remained indeterminate in 1 patient. The incidence rates of sludge occlusion, ingrowth occlusion, and overgrowth occlusion were 26.0%, 8.0%, and 2.0%, respectively. Successful transpapillary reintervention for RBO was performed in 94.7% (18/19) of patients, balloon cleaning was performed in 13 patients, and 5 patients required additional stent placement. One patient exhibited duodenal obstruction at reintervention, for which endoscopic ultrasound-guided hepaticogastrostomy was performed. The median time to RBO was 241 (95% confidence interval, 134-not applicable) days (Fig. 3). The median duration of overall survival was 175 (95% confidence interval, 115–281) days (Fig. 4).

## Discussion

This study demonstrated that DBS with endobiliary RFA possessed good technical feasibility and yielded good long-term outcomes and acceptable AE rates, especially a low ingrowth occlusion rate, for the management of MDBO.

Covered and uncovered SEMSs have their respective advantages and disadvantages. Covered SEMSs can prevent ingrowth, but are susceptible to sludge formation and stent migration, in addition to cholecystitis due to cystic duct obstruction^3,4^. The advantage of uncovered SEMSs is that they are not beset by issues such as cystic duct obstruction and migration, although occlusion due to ingrowth is a frequent occurrence. It is necessary to consider the major factors leading to RBO in each type of SEMS to achieve a longer stent patency period. Attempts have been made to develop covered SEMSs with anti-migration and anti-reflux functions^11,12^. The DBS was invented with the objective of reducing ingrowth, while preserving the advantages of uncovered stents. Lee et al.^5^ compared the DBS and conventional uncovered SEMS and reported that tumor ingrowth was less frequent and stent patency was longer with the DBS, indicating that it is a promising option, at least when choosing uncovered SEMSs.

Endobiliary RFA was first developed to extend the duration of stent patency. Some studies have shown that the stricture diameter can be improved after ablation^13,14^, which is considered to be one of the bases for the extension of the stent patency effect. Since RFA can prevent ingrowth or hyperplasia, it is assumed that it would have good compatibility when used in combination with the uncovered SEMS rather than the covered SEMS or plastic stent. Although a robust consensus has not been reached, some studies have shown prolonged stent patency when RFA is used in combination with the uncovered SEMS^6,15,16^. In this study, we hypothesized that ingrowth could be further reduced by using DBS in conjunction with RFA. Consequently, the ingrowth rate was low at 8.0%, which seems to be a promising result.

This study should be considered in the context of the limitations associated with its single-center setting and non-randomized design. Because of the lack of control group, whether the favorable outcome was contributed from both DBS and RFA or one independently cannot be answered with this study. Additionally, the results may not be directly applicable to other institutions because all procedures were performed or supervised by a single endoscopist experienced in performing endobiliary RFA and biliary stenting. Since observation is possible only under fluoroscopy during RFA, the ablation effect may depend on the experience of each endoscopist. Further multicenter prospective studies are needed to assess the utility of the combination of DBS and RFA and confirm the findings of the present study while comparing to other approaches.

In conclusion, this is the first study to evaluate the feasibility of the combination of DBS and RFA for the treatment for unresectable MDBO with favorable results. The two modalities demonstrated good compatibility and may serve as an effective option to reduce ingrowth, while preserving the benefits of the uncovered SEMS.

## Supplementary Information

Supplementary Video 1.

Supplementary Legends.

## Data Availability

The datasets used and/or analyzed during the current study available from the corresponding author on reasonable request.
