# Supplementary material for: Double bare metal stent deployment combined with intraductal radiofrequency ablation for malignant distal biliary obstruction: a prospective pilot study
Source: Sci Rep. 2023 Feb 14;13:2654. doi: 10.1038/s41598-023-29955-5 (PMC9929070; doi:10.1038/s41598-023-29955-5)
Supplement: Supplementary file 2 — Supplementary Legends. [file 41598_2023_29955_MOESM2_ESM.docx]

**Video 1**

Double bare uncovered metal stent deployment combined with intraductal radiofrequency ablation for a patient with malignant distal biliary obstruction
